# Causal Mediation Analysis in the Presence of Post-treatment Confounding Variables: A Monte Carlo Simulation Study

**DOI:** 10.3389/fpsyg.2020.02067

**Published:** 2020-08-14

**Authors:** Yasemin Kisbu-Sakarya, David P. MacKinnon, Matthew J. Valente, Esra Çetinkaya

**Affiliations:** ^1^Department of Psychology, Koç University, Istanbul, Turkey; ^2^Department of Psychology, Arizona State University, Tempe, AZ, United States; ^3^Center for Children and Families, Department of Psychology, Florida International University, Miami, FL, United States

**Keywords:** mediation, causality, g-estimation, propensity score, sequential ignorability

## Abstract

In many disciplines, mediating processes are usually investigated with randomized experiments and linear regression to determine if the treatment affects the outcome through a mediator. However, randomizing the treatment will not yield accurate causal direct and indirect estimates unless certain assumptions are satisfied since the mediator status is not randomized. This study describes methods to estimate causal direct and indirect effects and reports the results of a large Monte Carlo simulation study on the performance of the ordinary regression and modern causal mediation analysis methods, including a previously untested doubly robust sequential g-estimation method, when there are confounders of the mediator-to-outcome relation. Results show that failing to measure and incorporate potential post-treatment confounders in a mediation model leads to biased estimates, regardless of the analysis method used. Results emphasize the importance of measuring potential confounding variables and conducting sensitivity analysis.

## Introduction

Mediation analysis allows researchers to investigate the underlying mechanisms of a treatment and to address competing explanations. In a typical mediation model, an independent variable (*X*) causes a mediator (*M*), and then the mediator causes an outcome (*Y*). For example, a randomized health promotion program (*X*) may influence healthy eating (*Y*) via changing the dietary social norms (*M*) (Ranby et al., 2011). However, even though mediation analysis investigates causal mechanisms and involves causal inference by definition, most current mediation analysis methods rely on assumptions that may not be satisfied for causal conclusions (Holland, 1988; Kenny, 2008; MacKinnon, 2008; Stone-Romero and Roposa, 2008; Mayer et al., 2014). When treatments (i.e., variable *X*) are randomized in a mediation study, causal claims can be made for the effect of the treatment on the mediator because randomization balances confounders between groups and thereby reduces the possibility of confounders (i.e., extraneous variables that correlate with both the treatment and mediator). Similarly, randomization to conditions allows for a causal estimation of the treatment effect on the outcome variable. However, except for the experimental design options such as double randomization, individuals usually cannot be randomized to the level of the mediator because their score on the mediator is a result of their response to the treatment. Therefore, in a mediation context, a randomized treatment does not ensure accurate causal estimation of the relation between the mediator (*M*) and the outcome (*Y*). The path relating *M* to *Y*, adjusted for *X* and the path from *X* to *Y* adjusted for *M* (i.e., the direct effect) are still subject to potential confounding variables, as noted by many researchers (Holland, 1988; Pearl, 2001; MacKinnon, 2008; Imai et al., 2010a; MacKinnon et al., 2012).

The problem of causal inference in mediation led researchers to consider the causal assumptions of mediation analysis (Pearl, 2001; VanderWeele and Vansteelandt, 2009; Imai et al., 2010a, b) and suggest several methods for an accurate estimation of mediation in the presence of confounding variables. However, most of the recent literature on causal mediation has been theoretical or if a simulation study is included, it is small with a limited number of conditions and few path effect sizes (Lynch et al., 2008; Lepage et al., 2012; Ten Have and Joffe, 2012). The purpose of this paper is to address the research question of how robust the modern causal mediation methods are to violation of the no confounders assumption in a large simulation study and provide recommendations to researchers. The simulation study also includes a method – the doubly robust sequential g-estimation – that has been suggested in the literature to perform well but has never been, to our knowledge, described in detail nor evaluated (Vansteelandt and Keiding, 2011). Specifically, we compare the statistical performance of five analysis methods: regression with adjustment, inverse propensity weighting, inverse propensity weighting with truncated weights, sequential g-estimation, and doubly robust sequential g-estimation. We investigate the effect of confounder effect size, type of confounders (i.e., baseline vs. post-treatment), and violation of the assumption of no unmeasured confounders on the *M* to *Y* relation on the accuracy of indirect effect estimates in a single mediator model with a randomized *X*. Furthermore, based on the results of the simulation study, we recommend and discuss the importance of sensitivity analysis and experimental designs that involve the manipulation of the mediator whenever it is feasible.

### Linear Regression Approach to Mediation

**FIGURE 1:**
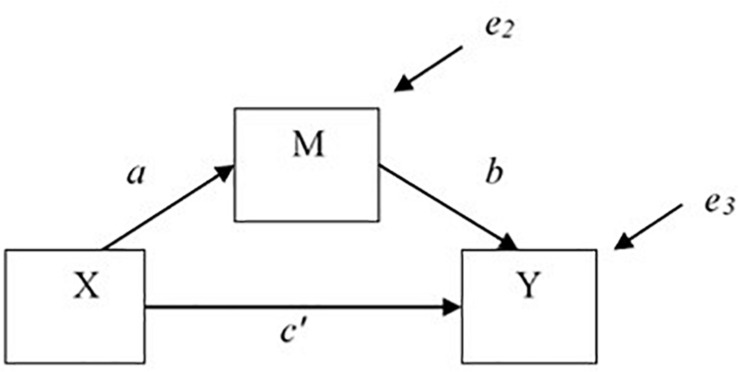
Single mediator model.

The most common approach to mediation employs Ordinary Least Squares (OLS) regression or structural equation modeling (Baron and Kenny, 1986; MacKinnon, 2008). The basic mediation model involves three equations including the following variables: *X*, the treatment variable, *Y*, the dependent variable, and *M*, the mediator (MacKinnon and Dwyer, 1993) (see Figure 1):

(1) E(Y|X)=i+1cX

(2) E(M|X)=i+2aX

(3) E(Y|X,M)=i+3c′X+bM

Equation 1 gives the expected *Y* given *X*, where *X* can take on values *x.* In other words, it estimates the *total effect* of the treatment *X* on outcome *Y* (the *c* regression coefficient). Equation 2 predicts the effect of *X* on the mediator (the *a* path). Equation 3, where *X* can take on values *x* and *M* can take values *m*, estimates the effect of treatment *X* on the outcome *Y* adjusting for the effects of the mediating variable *M* (the *c*′ path is the *direct effect* of *X* on *Y*) and the effect of *M* on *Y* adjusted for *X* (the *b* path). *i*_1_, *i*_2_, and *i*_3_ are intercepts; and *e*_1_, *e*_2_, and *e*_3_ are errors that are assumed to be independent across equations. The point estimate of the indirect effect is usually computed as the product of coefficients, *ab*, that is equal to the difference between the total and direct effects, *c* − *c*′ (i.e., the difference in coefficients method to compute the total indirect effect), in linear models with no missing data. The *ab* estimate of the indirect effect can then be divided by its standard error and this ratio can be compared to the normal distribution or other methods can be used to test for statistical significance (MacKinnon et al., 2002; Kisbu-Sakarya et al., 2014; Fritz et al., 2015). Note that lower case letters, *x*, *m*, and *y* represent values of variables *X*, *M*, and *Y*, respectively. This distinction between the variables and the values of the variables defines causal effects at different values, *X* = *x*, *M* = *m*, and *Y* = *y*, and allows for the possibility that different causal effects may be obtained at different values, *x*, *m*, and *y* of variables *X*, *M*, and *Y*, respectively.

Mediation analysis by linear regression has several assumptions. First, it is assumed that there is no measurement error that may cause bias in the estimators. It is also assumed that the variables are continuous and residual variance is normally distributed. Another assumption is that the causal paths between *X*, *M*, and *Y* have the correct functional form and do not have bidirectional effects (MacKinnon, 2008). Another critical assumption about causality is that there are no omitted variables affecting the causal relations in the mediation model, which will be described in detail in the following section (Holland, 1988; Robins and Greenland, 1992; Pearl, 2001, 2012).

### Potential Outcomes Approach to Mediation

The linear regression approach to compute the mediated effect as *ab* is based on observed values of the mediator and outcome. Another framework to define effects is the potential outcomes approach. The potential outcomes approach provides a basis for causal inference methods, including the ones used in causal mediation analysis. This approach to causal effects (Rubin, 1974, 2004, 2005; Holland, 1986, 1988; Morgan and Winship, 2014) defines the individual causal effect using the potential outcomes of the same individual. Starting with the individual level causal effect, let variable *X* be a treatment program with level *x* (*x* = 1 for the treatment, *x* = 0 for the control) and variable *Y* the outcome variable. An individual *i* may be assigned to the treatment group (*x* = 1) and obtain the potential outcome value *Y*_i_(1). The second potential outcome for that individual in the treatment group is the value she would have obtained on the outcome variable if she had been assigned to the control condition (*x* = 0), that is *Y*_i_(0) (also referred to as the counterfactual value). The corresponding individual causal effect is then equal to the difference between the potential outcomes, *Y*_i_(1) − *Y*_i_(0). However, because it is often not possible to observe both outcomes for the same person the individual causal effect cannot be computed. This is referred to as the “fundamental problem of causal inference” by Holland (1988). To overcome this challenge, averages of individuals are used to compute the average causal effect, *E*[*Y*_i_(1) − *Y*_i_(0)]. This average causal effect solves the problem of estimating causal effects for each individual. The average causal effect, the difference between the means in the treatment and control groups, is a causal effect when individuals are randomized to conditions and the randomization has been successful.

The potential outcomes approach provides a new framework to interpret mediation effects. In the case of a single mediator model, let *Y*_i_(*x*, *m*) denote the potential outcome for an individual under the treatment level *x* and mediator level *m*. Let *X*_i_ be a binary treatment variable (*x* = 0 for the control group, and *x* = 1 for the treatment group). The counterfactual value for the continuous mediator is denoted as *m*′. This single mediator model gives rise to the formulation of the following effects: controlled direct effect, natural direct effect and natural indirect effect (Robins and Greenland, 1992; Pearl, 2001, 2009).

#### Natural and Controlled Effects

##### Definition

The controlled direct effect is the effect of *X* on *Y* at a specific value *m* of *M*. More formally, the controlled direct effect (CDE) of a treatment on the outcome is the difference between the potential outcome scores when the individuals’ mediating variable score was controlled and set to a specific value (Robins and Greenland, 1992).

(4) CDE=E[Y(1,m)i-Y(0,m)i].

As opposed to the controlled direct effect, *natural direct effects* (NDE) are the effects of the treatment on outcome when fixing the level of the mediator to one of its potential values and changing the level of *X*:

(5) NDE=E[Y(1,M)xi-Y(0,M)xi].

Similarly, *average natural indirect effects* are the effects of the treatment on outcome when changing the level of the mediator to one of its potential values under a fixed value of *X*:

(6) NIE=E[Y(X,iM)i1i-Y(X,iM)i0i]

The natural direct and indirect effects add up to the total effect, when the *X* and *M* interaction is assumed to be zero. When the *X* and *M* interaction is non-zero, Pearl (2001) demonstrated that the sum of the natural direct and indirect effects equal to the total effect (TE) (i.e., TE − NDE = NIE) at selected levels of *X* and, importantly, this result also holds in models with non-linear effects such as logistic regression. MacKinnon et al. (2020) illustrates the correspondence between potential outcomes and traditional estimators of the indirect effect when the *X* and *M* interaction is non-zero, in the form of simple direct and indirect effects.

##### Identification

The four assumptions below are required for the natural effects to be identified (Pearl, 2001; VanderWeele and Vansteelandt, 2009; VanderWeele, 2010a, 2011).

(i) No unmeasured confounder for the relation between *X* and *M.*
(ii) No unmeasured confounder for the relation between *X* and *Y*.
(iii) No unmeasured confounder for the relation between *M* and *Y*.
(iv) No *M* to *Y* confounder affected by treatment.

Confounding occurs when there are common causes (i.e., confounders) of the independent variable and the dependent variable. Omitting a confounder from a statistical model may lead to biased estimates. Assumptions (i) and (ii) refer to the ignorability of treatment assignment conditional on the observed baseline confounders. Assumption (iii) refers to the ignorability of the mediator conditional on the observed treatment and pretreatment confounders. The linear regression approach to mediation assumes sequential ignorability, which involves the ignorability of the treatment assignment and the ignorability of the mediator. Assumptions (i) and (ii) are frequently fulfilled with randomization of the treatment *X*. Assumption (iii) means that there are no unmeasured confounders influencing the *b* path. This assumption usually fails because the mediator status is not randomly assigned, but rather naturally occurs under the assigned treatment condition. Even though we may condition on observed confounders for the relation between *M* and *Y*, unobserved confounders can still exist. Unobserved confounders may be present in many studies which compromises the causal interpretation of both *c*′ and *b* coefficients, thus the direct and indirect effects (MacKinnon, 2008, Chapter 13; MacKinnon et al., 2013).

Identification of the indirect effect using the controlled effect approach requires assumptions i, ii, iii, and no interaction between *X* and *M* assumption (assumption v). When there is no interaction between *X* and *M*, the CDE becomes equal to NDE which then allows for the computation of the indirect effect again as the difference between the total effect and the CDE. The computation of the indirect effect using this approach assumes normally distributed variables in addition to the no XM interaction assumption. Note that identification of CDE only requires assumptions ii and iii. Additionally, all effects assume that the potential outcomes for an individual do not depend on the treatment assignment or mediator level of other individuals (i.e., no interference between individuals).

Several solutions to estimate controlled effects have been proposed to improve the accuracy of the *b* and *c*′ coefficients as causal estimates, and correspondingly interpretation of indirect effect as a causal effect (Ten Have et al., 2007; Vansteelandt, 2009; VanderWeele and Vansteelandt, 2009; Ten Have and Joffe, 2012). Below, we describe some of those methods we investigate in our simulation study. One should note that the methods differ in how they control for confounders and assumptions. Therefore, researchers should pay attention to which effects they are interested in estimating and the assumptions made by the analysis method they choose.

##### Estimation

###### Inverse propensity weighting method

Returning to the case of one *X* and one *Y* variable, the causal effects of a non-randomized treatment on an outcome can be estimated using propensity scores that account for the effects of potential confounders of the *X* to *Y* relation. In this section, estimation using propensity scores is described first followed by the use of propensity scores in causal mediation.

###### Propensity scores

In the case of an effect of treatment on the outcome, the propensity score is the estimated probability of receiving the treatment given measured confounders (Rosenbaum and Rubin, 1983). Because the confounders used to estimate the propensity score are either variables that do not change, such as gender, or variables measured at baseline, the estimated propensity scores are not influenced by the treatment. Therefore, assuming all confounders are measured, comparing the treatment and control groups with similar estimated propensity scores is a causal estimator of the unconfounded effect of *X* on *Y*. In other words, propensity scores balance the distribution of confounders in the treatment and control groups so that the treatment assignment effect on the outcome is unconfounded given the propensity scores. An advantage of using propensity scores over analysis of covariance as a method to adjust for confounders is that including a large set of confounders in an analysis of covariance model is sometimes not practical whereas the propensity score is a single number summarizing all of the measured confounders. Moreover, propensity score methods allow the researcher to easily assess if the distributions of confounders in the treatment and control groups overlap adequately (Rubin, 1997; King and Zeng, 2006), whereas this assumption is not easily visualized or assessed in an ANCOVA. If there is not adequate overlap, propensity score methods are not used. This leads the propensity score method to estimate more stable treatment effects, as compared to ANCOVA. There are several propensity score methods for confounder adjustment; among them are matching (Rosenbaum and Rubin, 1985; Rubin and Thomas, 1992, 1996), stratification (Rosenbaum and Rubin, 1984), and weighting (Robins et al., 1995; Hirano and Imbens, 2001). Here, we focus on a weighting method called inverse propensity weighting (IPW) to improve causal inference in the case of confounders affecting the *M* to *Y* relation in the single mediator model. Note that we don’t keep the *i* subscript in the next sections for simplicity.

###### Creating propensity scores and weighting in the mediation context

For a non-randomized treatment effect on an outcome, inverse propensity weighting makes the treated and control participants represent the population by weighting each observation. The weights reflect the probability that each person would have received the treatment based on measured pre-treatment confounders. The weights are the inverse of the probability of being in the group that an individual actually participated in, conditional on the confounders (*C*). In other words, individuals in the treatment group are weighted by 1/*P*[*X* = 1— *C* = *c*] and individuals in the control group are weighted by 1/(1 − *P*[*X* = 1—*C* = *c*]). In this framework, the causal inference challenge is viewed as a missing data problem (Robins et al., 1994), in that *Y*_i_(1) is only observed for individuals under the treatment condition and is missing for the individuals in the control group. Inverse weighting works as a strategy to account for the counterfactual values of the outcome scores.

Propensity scores can be used to improve the causal interpretation of the indirect effects in a similar way as for the *X* to *Y* effect. If the mediator is binary with values of 0 and 1, then individuals with *M* = 1 are given a weight of *P*[*M* = 1— *X* = *x*]/*P*[*M* = 1— *X* = *x*, *C* = *c*]. And individuals with *M* = 0 are given a weight of (1 − *P*[*M* = 1— *X* = *x*])/(1 − *P*[*M* = 1— *X* = *x*, *C* = *c*]). In the mediation context, the confounders used for weighting are measured before the mediator. The weights reflect the additional prediction of the confounders compared to the prediction by treatment alone. The purpose of these weights is to create a new data set in which confounding by measured variables is removed so that the relation of *M* to *Y* more closely resembles a randomized relation. For a binary mediator, the denominator model can be computed by a logistic regression of the mediator on measured confounders and the treatment condition. The predicted probabilities are the propensity score estimates (denoted as $\hat{\pi }$). If the mediator is continuous, then the denominator model can be computed by regressing the mediator on measured confounders and the treatment and then inserting the predicted values ($\hat{\text{m}}$) in a normal probability density function (Robins et al., 2000; Coffman and Zhong, 2012) as shown below:

(7) $$\phi \,\left(M|X,C\right)=\frac{1}{\sqrt{2\,\pi \,\sigma^{2}}}\,e^{-\frac{{\left(m-\hat{\text{m}}\right)}^{2}}{2\,\sigma^{2}}}$$

where σ is the residual standard error from the regression of *M* on *X* and *C*.

###### Estimating the indirect effect

Two marginal structural models (MSM) equations (equations 8 and 9) are used to define mediated effects (VanderWeele and Vansteelandt, 2009; Coffman and Zhong, 2012):

(8) E[M|x]=i+0⁢Max

which defines the effect of X on M as:

E[M(1)-M(0)]=(i+0⁢Ma1)-(i+0⁢Ma0)=a

and

(9) E[Y|m,x]=i+0⁢Ybm+c′x

which defines the effect of the continuous *M* (for which a reference value is indicated as *m*′) on *Y* for *x* = 1 as:

E[Y(1,m)−Y(1,m′)]       =(i+0Ybm+c′)−(i+0Ybm′+c′)=b(m−m′)

and the effect of the continuous *M* on *Y* for *x* = 0 as:

E[Y(0,m)−Y(0,m′)]       =(i+0Ybm)−(i+0Ybm′)=b(m−m′).

Thus, *b* defines the causal effect of one unit increase in *M* on *Y*, at each level of the treatment (Coffman and Zhong, 2012).

If the treatment in the mediation model is randomized, then only equation 9 is weighted using the propensity scores. If the treatment is not randomized, then equation 8 (the effect of *X* on *M*) should also be weighted. The null hypothesis stating that the product of the *a* and *b* paths is equal to zero can be tested to assess mediation (Coffman and Zhong, 2012).

A possible problem in propensity weighting is the presence of extreme weights. Extreme variation in the weights can yield high variance and instability in the estimates. A solution to reduce the impact of extreme weights is weight truncation (Potter, 1993). Weight truncation is generally performed by trimming the weights that are larger or smaller than some values (e.g., cut points at the 1st or 99th percentile of the weight distribution). Yet, simulation studies show that even though weight trimming can improve the performance of propensity score weights in some conditions, it can also induce bias in other conditions (Lee et al., 2011). Therefore, researchers are advised to use weight trimming with caution and focus more on improving the specification of the propensity score model as compared to methods such as trimming (Lee et al., 2011).

###### Sequential g-estimation

G-estimation is a method to identify the controlled direct effect in the presence of post-treatment confounders (Ten Have et al., 2007; Ten Have and Joffe, 2012). Post-treatment confounders in a mediation model are confounders of the *M* to *Y* relation that are influenced by the treatment; they can bias the direct effect (i.e., *c*′) estimate. An example of post-treatment confounders for the *M* to *Y* relationship may be the variable socio-economic status (SES) in a mediation chain where educational attainment influences unhealthy eating behavior, which then influences blood pressure. In this example, SES may be influenced by educational attainment and also influence both eating behavior and blood pressure. Another example of post-treatment confounders in mediation may be alliance with the therapist in a cognitive therapy intervention to decrease work-related stress through enhancing coping skills. In this example, alliance with the therapist is not a mediator that is targeted by the stress management program and instead may even bias the indirect effect estimate as a post-treatment confounder.

A method to handle a post-treatment confounder is the g-computation method that attempts to estimate all potential values in a research design by using the estimated distribution of the measured confounders given values of *X* (Robins, 1986; Ten Have et al., 2007). However, the g-computation method can be difficult to implement when estimating the joint distribution of the confounders as a function of treatment in the case of many confounders, since the method requires estimating all predicted potential outcomes. A simpler method is sequential g-estimation, which is equivalent to g-computation method in the case of linear models, which allows the researchers to directly model the effect of treatment on the outcome (Goetgeluk et al., 2008; Joffe and Green, 2009; Vansteelandt, 2009).

The sequential g-estimator is implemented in two steps in which the first step removes the effect of the mediator from the outcome variable and in the second step the direct effect is estimated. First, the outcome is regressed on the treatment, mediator, and post-treatment confounders using OLS regression to find the mediator’s effect on the outcome (this is referred to as the mediator model). Then, the mediator’s effect is removed from the outcome by using the coefficient reflecting the effect of *M* on *Y*, (*Y* − β_m_ *M*). Next, this residual outcome is regressed on the treatment to find the remaining direct effect of *X* on *Y* (this is referred to as the outcome model):

(10) E(Y-βMm|X)=α+0ψX

The indirect effect can then be computed as the difference between the total effect and the CDE. The above equation for the residual outcome can also include the baseline confounders, but not post-treatment confounders. The standard error for the sequential g-estimator, ψ, may be biased because it does not account for the uncertainty in the estimation of the mediator’s effect. Therefore, bootstrapping can be used for the estimation of the standard error. Note that the g-estimator requires assumptions i, ii, iv, and v.

###### Doubly robust sequential g-estimation

Because the sequential g-estimation method fits two models in its estimation (by first estimating a mediator model and then an outcome model as described above), it may be biased by misspecification in either of these two models. A *doubly robust sequential g-estimation* method is suggested in the literature in which the estimated direct effect is robust to misspecifications in either the mediator or the outcome model (Vansteelandt and Keiding, 2011). The method is expected to produce bias in the direct effect estimates when both parts of its estimation process are misspecified (Schafer and Kang, 2008). Doubly robust sequential g-estimation involves the following steps: In the first step, a propensity model for the mediator is fitted as in the IPW method; then, in the second step, the outcome regression is fitted using the propensity weights. Even though this method to estimate the controlled direct effects has been recommended, its performance has never been tested in simulation studies.

## Simulation Study

Recent literature suggests various methods to deal with the assumption of no unmeasured confounders for the *M* to *Y* relation in mediation analysis. The methods differ in how the adjustment is made for confounders. Simulation studies show that the inverse propensity weighting approach produces roughly unbiased estimates of the indirect effects when all pre-treatment or post-treatment confounders are measured and included in the propensity model (Coffman and Zhong, 2012; Coffman et al., 2016). Similarly, sequential g-estimation has been shown to produce unbiased estimates of the direct effect in the case of including all post-treatment confounders in the estimation process, whereas linear regression with adjustment does not (Loeys et al., 2013). Additionally, sequential-g estimation produces roughly unbiased direct effect estimates as the association between the post-treatment confounder and the outcome increases. Yet, the adjusted regression and IPW estimators get increasingly biased as the association between the post-treatment confounder and the outcome increases (Robins et al., 2000; Vansteelandt, 2009). A recent simulation study also showed that the modern methods perform well when post-treatment confounders are observed, yet did not test the no unmeasured confounding assumptions and the effect of confounder effect size on bias (Coffman et al., 2016).

In this study, we aim to investigate the effect of confounder effect size, type of confounders (i.e., baseline vs. post-treatment), and violation of the assumption of no unmeasured confounders for the *M* to *Y* relation on the accuracy of indirect effect estimates produced by these modern methods using a large simulation design.

### Method

#### Simulation Overview

**FIGURE 2:**
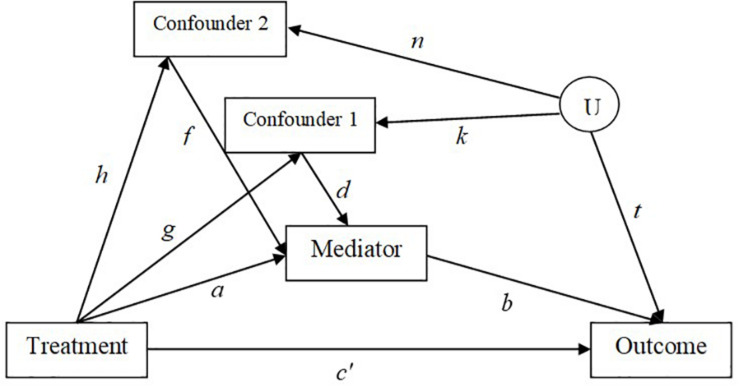
Generated model with mediator to outcome confounders.

A Monte Carlo simulation study was conducted to examine the effect of confounder effect sizes and violation of the assumption of no unmeasured confounders for the *M* to *Y* relation on the performance of five analysis methods (i.e., regression with adjustment, IPW, IPW with truncated weights, sequential g-estimation, and doubly robust sequential g-estimation) in a single mediator model with two confounder variables of the *M* to *Y* relationship. There are two measured confounder variables (*C*_1_ and *C*_2_) that influence the mediator directly, and the outcome through a spurious relation induced by an unobserved confounder U (see Figure 2). The generated model is based on Vansteelandt (2009). The adjustment for the collider *M* (i.e., a variable affected by two other variables in a causal diagram) along the path *X* to *M* to *C* to *U* to *Y* makes *X* and *Y* dependent along that path and may induce selection bias, especially when confounders are affected by the treatment (Goetgeluk et al., 2008; Vansteelandt, 2009). The model was generated with different effect sizes for the paths *X* to *M*, *M* to *Y*, *X* to *C*_1_, *X* to *C*_2_, *C*_1_ to *M*, and *C*_2_ to *M*. After the generation of the data, the five methods were used to estimate the indirect effect estimates in the single mediator model. To assess the effect of violation of the assumption of no unmeasured confounders, two models are estimated using the five methods: (a) a two-confounders estimation of the model by including both confounders (*C*_1_ and *C*_2_) in the estimation; (b) a one-confounder estimation of the model by including only the confounder *C*_1_ in the estimation and omitting the second confounder *C*_2_ from the estimation. The data were generated and analyzed in SAS 9.3 with a total of 1,000 replications per condition.

#### Data Generation and Simulation Conditions

The following regression equations are specified in SAS in order to generate the population parameters. Figure 2 shows the simulated model. Exogenous variables are generated using the SAS RANNOR function to produce normally distributed random variables. The independent variable *X* is simulated to be binary to represent a treatment status (0 = control, 1 = treatment group). All other variables are simulated to be continuous with normally distributed error terms. There is an unobserved confounder *U* in the simulated model so that there is only one path to be traced from *X* to *Y* for ease of interpretation.

(11.1) M=aX+dC+1fC+2e1

(11.2) $$Y=c^{\prime }X+bM+tU+e{}_{2}$$

(11.3) C=1gX+kU+e3

(11.4) C=2hX+nU+e4

The unstandardized regression parameters for the *b* and *c*′ paths are varied as 0,0.14, and 0.59. The effect of *X* on *M* (the *a* path), and the effects of *C*_1_ on *M*, and *C*_2_ on *M* (the paths *d* and *f*) are varied as 0.14,0.39, 0.59. The effect of *X* on *C*_1_ and *C*_2_ (the paths *g* and *h*) are varied as 0,0.14, and 0.59. The effects of *C*_1_ on *M* and *C*_2_ on *M* are set to be equal (i.e., the *d* and *f* paths), as well as the effect of *X* on *C*_1_ and *C*_2_ (i.e., the *g* and *h* paths). The effects of the unobserved confounder *U* on *C*_1_, *C*_2_, and *Y* (i.e., the paths *k*, *n*, and *t*) are set equal to 1.0. Mediation paths effect sizes were chosen following MacKinnon et al. (2002) to approximately correspond to small, medium, and large effect sizes (Cohen, 1988). Sample size was set to 500 in each condition. To summarize, a 3 (*X* → *M*) × 3 (*M* → *Y*) × 3 (*X* → *Y*) × 3 (*C*_1_ → *M* and *C*_2_ → *M*) × 3 (*X* → *C*_1_ and *X* → *C*_2_) factorial design yielded a total of 243 simulation conditions. Furthermore, in order to investigate models of mediation with paths *d* and *f* taking values of opposite signs, we have simulated the following conditions: *d* = −0.14 and *f* = 0.14; *d* = 0.14 and *f* = −0.14; *d* = −0.14 and *f* = −0.14; *d* = −0.59 and *f* = 0.59; *d* = 0.59 and *f* = −0.59; *d* = −0.59 and *f* = −0.59 when the *b* paths were set to 0 or to 0.59 resulting in 12 additional conditions. These conditions were only simulated when *g* and *h* were equal to *d*, *c*′ was equal to 0, and the *a* path was equal to 0.59. It should be noted that we ran an extra condition of zero confounding (i.e., the confounder effect sizes were equal to zero) as a simulation check, and in that case, all estimation methods including the linear regression produced accurate indirect effect estimates.

#### Model Estimation

The five methods were applied to the generated data sets using two model estimation specifications: (a) The two-confounders estimation model including both of the population model confounders *C*_1_ and *C*_2_ in the estimation, and (b) the one-confounder estimation model including only the confounder *C*_1_ in the estimation (i.e., omitting the second confounder *C*_2_ from the estimation). The case of a one-confounder estimation model allows a test of the robustness of methods to the violation of no omitted confounders assumption. An exception was that the one-confounder model for the doubly robust sequential g-estimation that had three types of estimation where *C*_2_ was included in one part of the model but not another part of the model as will be described below.

(1) *Linear regression with adjustment.* The linear outcome regression equation for the two-confounders estimation model includes *X*, *M*, and both *C*_1_ and *C*_2_ as predictors of *Y* when estimating *c*′; and the one-confounder estimation model only includes *X*, *M*, and *C*_1_ as predictors of *Y* when estimating *c*′.
(2) *Inverse propensity score weighting*. The two-confounders estimation for the propensity score model to create the weights for the mediator is specified by including *X*, *C*_1_ and *C*_2_ in estimating the denominator model. The one-confounder estimation was performed by only including *X* and *C*_1_ in estimating the denominator model. In both cases, the weighted outcome model only includes *X* and *M* as predictors.
(3) *Inverse propensity score weighting with truncated weights.* The model specification is the same as the method (2) described above; yet weights are truncated at the 1st and the 99th percentile of the weight distribution as in Cole and Hernán (2008). The truncation is conducted to avoid weighting certain observations too little or too much.
(4) *Sequential g-estimation.* The first step in which the outcome is regressed on the treatment, mediator, and confounders using ordinary least squares regression (referred to as the Q-model) is specified by including *X*, *M*, *C*_1_, and *C*_2_ as predictors in the two-confounders estimation model. Only *X*, *M*, and *C*_1_ are included in the one-confounder estimation model.
(5) *Doubly robust sequential g-estimation*. In the first step, the propensity model for the mediator is fitted as in method (2), the IPW method. Then, in the second step, the outcome regression is fitted using the propensity weights. For the doubly robust sequential g-estimation method, three one-confounder estimation models are fitted: (a) by omitting confounder *C*_2_ in only the mediator propensity model, (b) by omitting confounder *C*_2_ in only the outcome model, (c) by omitting confounder *C*_2_ in both the mediator propensity and outcome models. This allows for testing if the doubly robust method fails when either parts or one part of the estimated model omit the confounder *C*_2_ (Schafer and Kang, 2008).

#### Data Analysis, Outcome Measures, and Evaluation Criteria

The total indirect effect is computed as subtracting the direct effect from the total effect (*c* − *c*′). For all methods, the percentile bootstrap with 1,000 replications is used to calculate the 95% confidence intervals. *Bias* of the indirect effect *c* − *c*′ are defined as:

$$\text{Bias}\,\left({\hat{\theta }}_{\text{c}}\right)=R^{-1}\,\sum_{r=1}^{R}\left({\hat{\theta }}_{\text{rc}}-\theta_{\text{c}}\right)$$

where *R* refers to the total number of replications, θ_*c*_ refers to the true value of the estimate, and ${\hat{\theta }}_{\text{rc}}$ refers to the parameter estimate for replications *r* in condition *c*. Bias indicates whether the observed parameter estimate consistently over- or underestimates the true value of the estimate. Additionally, the mean square error (MSE) is defined as follows:

$$\text{MSE}=R^{-1}\,\sum_{r=1}^{R}{\left({\hat{\theta }}_{\text{rc}}-\bar{\hat{\theta }}\right)}^{2}$$

MSE is equal to the variance of the estimates plus the square of bias. Therefore, it takes both bias and precision into account to assess the accuracy of the estimator. A low MSE indicates that the estimate is closer to the true value due to high precision and/or small bias.

## Results

**FIGURE 3:**
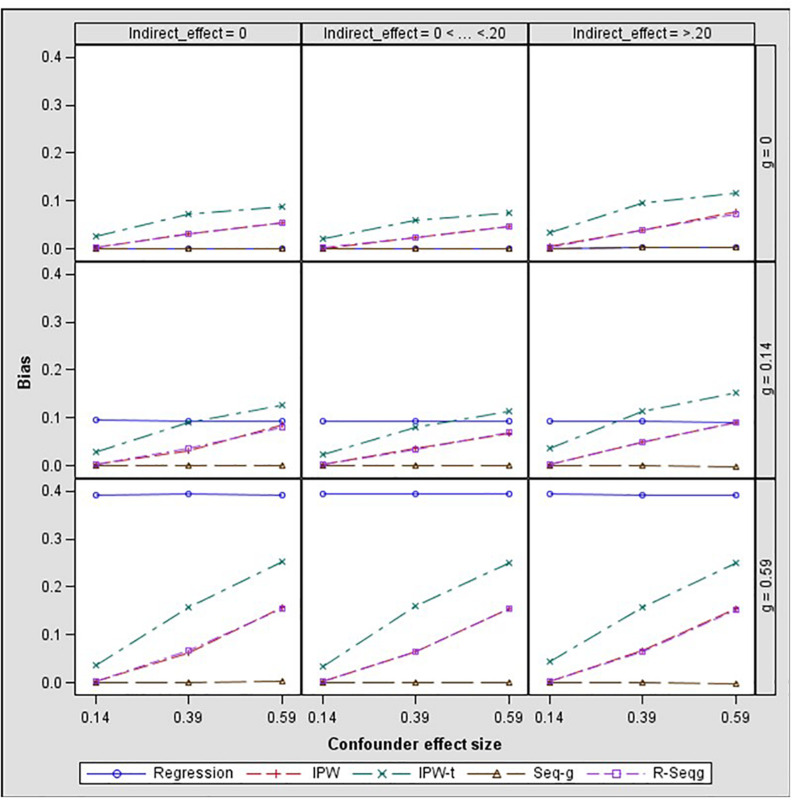
Bias in the indirect effect by confounder to mediator (*d* path) and treatment to confounder (*g* path) effect size – two-confounders estimation model. Regression, linear regression adjusting for covariates; IPW, inverse propensity weighting; IPW-t, IPW with truncated weights; Seq-g, sequential g-estimation; R-Seqg., doubly robust sequential g-estimation.

**FIGURE 4:**
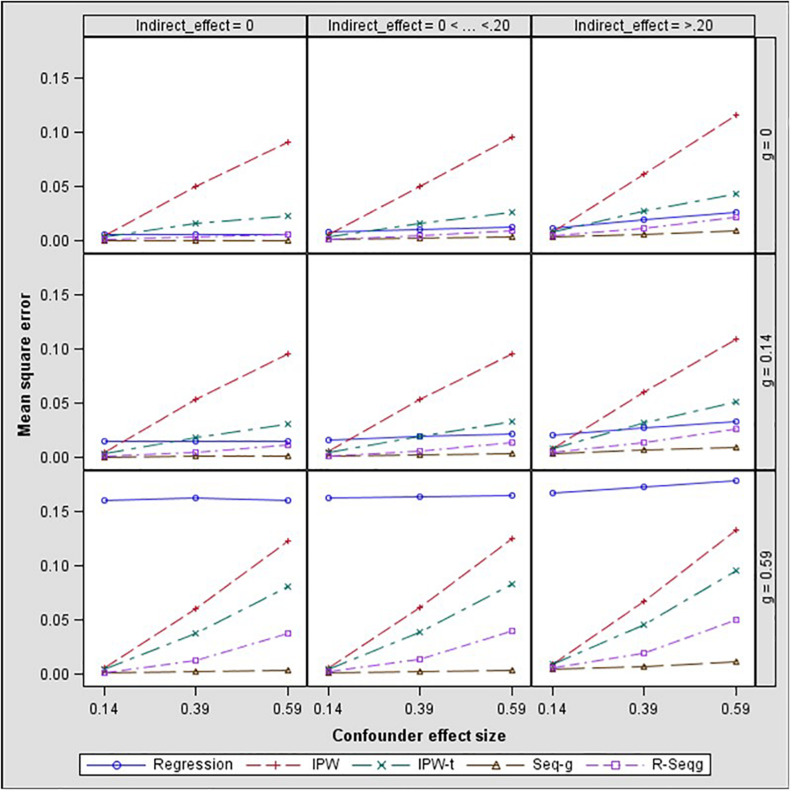
Indirect effect mean square error by confounder to mediator (*d* path) and treatment to confounder (*g* path) effect size – two-confounders estimation model. Regression, linear regression adjusting for covariates; IPW, inverse propensity weighting; IPW-t, IPW with truncated weights; Seq-g, sequential g-estimation; R-Seqg., doubly robust sequential g-estimation.

In Figures 3 and 4, bias and mean square error of estimates of indirect effects for two-confounders estimation models are presented across different effect sizes for the relation between the confounders and the mediator, i.e., paths *d* and *f* in Figure 2. The first rows of the Figure panels present the results when the confounders are not influenced by the treatment (i.e., the *g* and *h* paths in Figure 2 being equal to zero), and the second and third rows of figure panels present the results for the case of post-treatment confounders.

Figure 4 shows that when the confounders are at baseline, IPW methods have increasing bias as the confounder effect size increases while sequential g-estimation and linear regression with adjustment perform well. When the size of the indirect effect is larger than 0.20, the magnitude of the bias produced by the IPW-truncated method is approximately 0.10 which means 50% relative bias for a 0.20 true value of the indirect effect estimate. This indicates a large bias since an estimator can be considered as acceptable in terms of bias if the absolute value of relative bias is less than 10% (Flora and Curran, 2004). In the case of measured post-treatment confounders, sequential g-estimation performs the best in terms of bias and MSE across all confounder effect sizes. The doubly robust sequential g-estimation method has increasing bias as the post-treatment confounder effect size increases. This finding may be expected considering that the doubly robust g-estimation method uses IPW in its estimation process, and the IPW method produces more biased estimates as the confounder effect size increases. Results show that weight trimming does not improve the performance of the IPW method in terms of bias but this finding may be due to the trimming rule used. The optimal level of trimming may be difficult to determine and may not contribute to or have adverse effects in the estimation. On the other hand, IPW with truncated weights has a lower mean squared error (MSE) than the IPW as expected.

**FIGURE 5:**
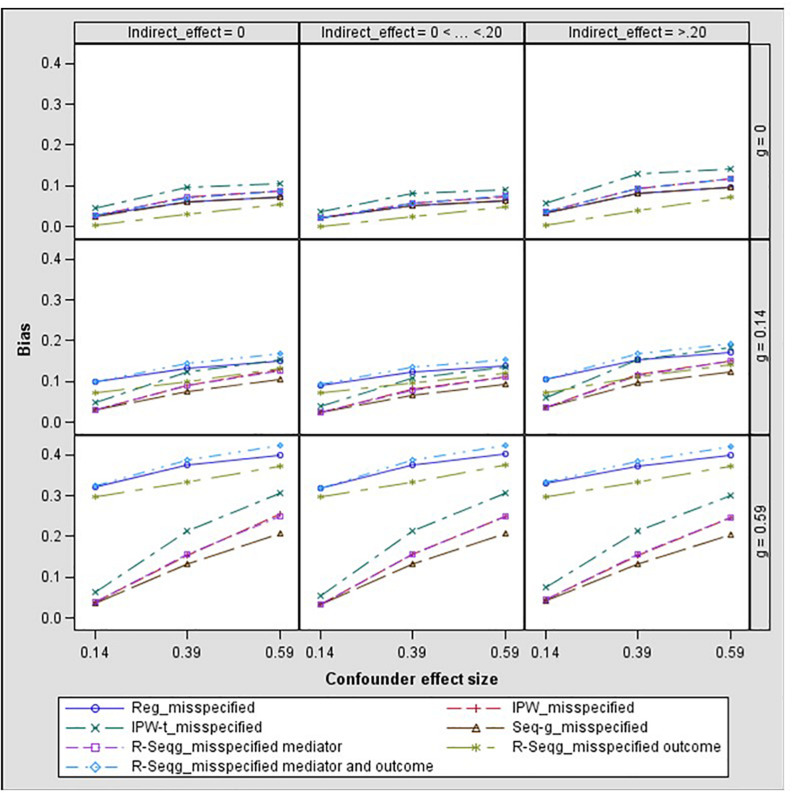
Bias in the indirect effect by confounder to mediator (*d* path) and treatment to confounder (*g* path) effect size – one-confounder estimation model. Reg, linear regression adjusting for covariates; IPW, inverse propensity weighting; IPW-t: IPW with truncated weights; Seq-g, sequential g-estimation; R-seqg_misspecified mediator, doubly robust sequential g-estimation with one-confounder estimation mediator model; R-seqg_misspecified outcome, doubly robust sequential g-estimation with one-confounder estimation outcome model; R-seqg_misspecified mediator and outcome, doubly robust sequential g-estimation with one-confounder estimation mediator and outcome models.

**FIGURE 6:**
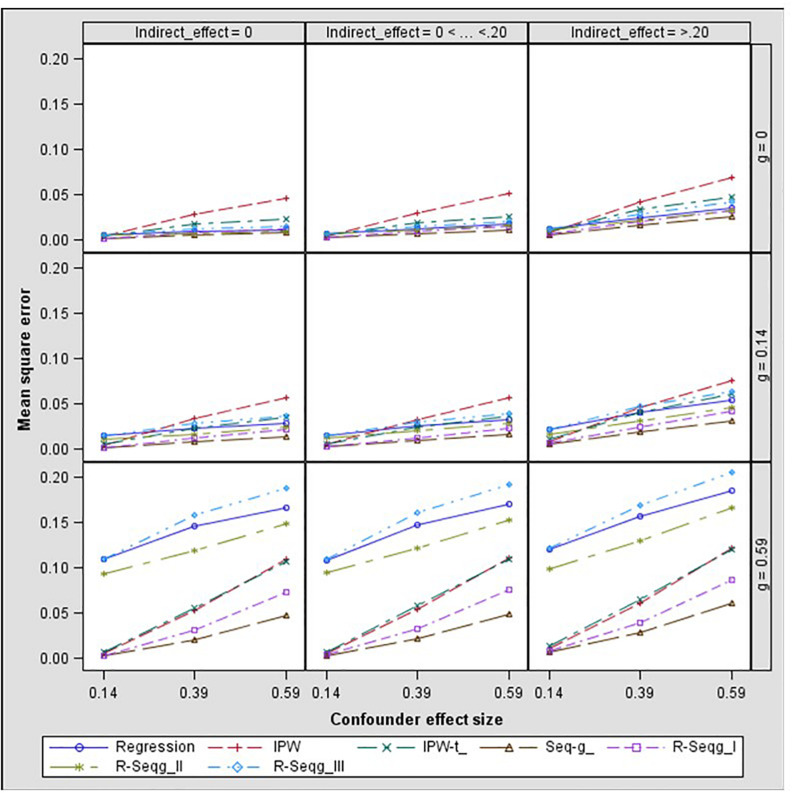
Indirect effect mean square error by confounder to mediator (*d* path) and treatment to confounder (*g* path) effect size – one-confounder estimation model. Reg, linear regression adjusting for covariates; IPW, inverse propensity weighting; IPW-t, IPW with truncated weights; Seq-g, sequential g-estimation; R-seqg_misspecified mediator, doubly robust sequential g-estimation with one-confounder estimation mediator model; R-seqg_misspecified outcome, doubly robust sequential g-estimation with one-confounder estimation outcome model; R-seqg_misspecified mediator and outcome, doubly robust sequential g-estimation with one-confounder estimation mediator and outcome models.

Figures 5, 6 present the results for one-confounder estimation models where one of the confounders, *C*_2_, was omitted from the analyses. This model with *C*_2_ omitted from the analysis corresponds to the common case in mediation studies in which no measure of a confounder is available, but a confounder may affect the analysis. A salient result from all one-confounder estimation models is that omitting an existing confounder for the *M* to *Y* relationship leads to increasing levels of bias as the effect of the treatment on the confounder increases. Especially when the effect of treatment on the confounder is large, then all methods have severe bias and MSE for the case of large confounder effect size. The results also show that doubly robust sequential g-estimation performs the worst when both the mediator and the outcome estimation models violate the no omitted variables assumption.

**FIGURE 7:**
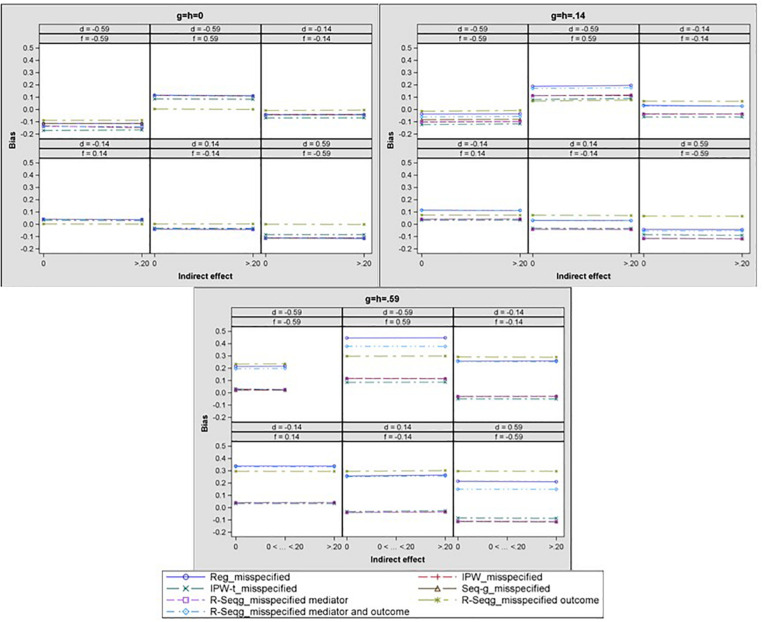
Bias in the indirect effect by confounder 1 to mediator (*d* path), confounder 2 to mediator (*f*), and treatment to confounder (*g* path) effect size – one-confounder estimation model. Reg, linear regression adjusting for covariates; IPW, inverse propensity weighting; IPW-t, IPW with truncated weights: Seq-g, sequential g-estimation; R-seqg_misspecified mediator, doubly robust sequential g-estimation with one-confounder estimation mediator model; R-seqg_misspecified outcome, doubly robust sequential g-estimation with one-confounder estimation outcome model: R-seqg_misspecified mediator and outcome, doubly robust sequential g-estimation with one-confounder estimation mediator and outcome models.

In order to investigate models of mediation that applied researchers can confront in real life, we also simulated the case of confounders with opposite signs (e.g., paths *d* and *f* taking values of 0.14 and −0.14). Since the misspecified models in which some confounders are omitted from the analyses depict the majority of real-life cases, we have only focused on these models for these conditions. Figure 7 displays the bias in the mediated effect for the misspecified models that did not contain confounder *C*_2_ in the estimation. Results show that when the models were misspecified, and one or both of the effects of the confounders on the mediator were negative, a similar pattern emerged compared to when both of these effects were positive with one exception. That is, these models had less bias when the effects of the confounders on the mediator were both large and negative and the effect of treatment on the confounders was large and positive compared to small and positive.

## Illustrative Example

We illustrate the application of causal mediation methods using an example based on a recent study in the Journal of Occupational and Organizational Psychology. Kovjanic et al. (2013) conducted a randomized experiment to investigate the effect of transformational leadership techniques on persistence as mediated by worker’s need for autonomy. A review by Judge et al. (2006) suggests that transformational learning is positively associated with factors such as commitment to the workplace and organizational identification. Because of the positive association of transformational learning with these factors that are not worker’s psychological needs, it is possible these factors could act as potential post-treatment confounders of the transformational learning experimental manipulation which are associated with the mediator and outcome.

**FIGURE 8:**
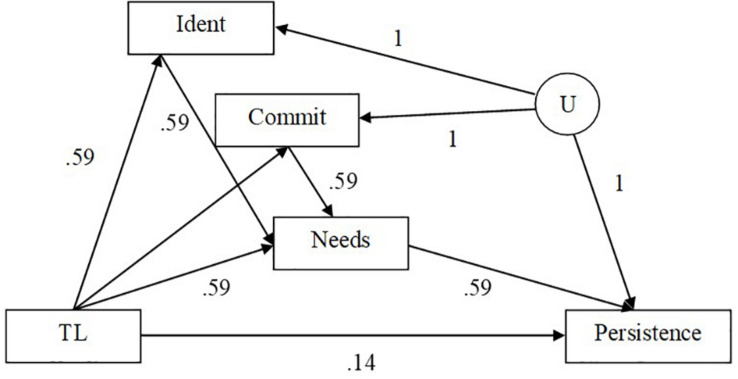
Illustrative example of mediated effect transformational leadership (TL) on persistence through its effect on need for autonomy (Needs) with post-treatment variables, commitment to workplace (Commit) and identification (Ident) and unmeasured confounder, *U*.

Figure 8 shows the model that corresponds to the example with transformational leadership (TL) as the predictor, need for autonomy as the mediator (Need), persistence as the outcome, and commitment to the workplace (Commit) and organizational identification (Ident) as post-treatment confounders. The mediated effect was estimated using the models that were investigated in the simulation study of this paper. As real data on confounders were not available, we have simulated data using the parameter values in Figure 8 for *N* = 500. Parameter values have been chosen from our simulation study. Across all models and whether or not the models were misspecified, the mediated effect was statistically significant as indicated by zero not being within the 95% percentile bootstrap confidence intervals.

Regarding the illustrative example, if a researcher was going to estimate the mediated effect of transformational leadership on persistence of the worker through its effect on the psychological needs of the worker and there were post-treatment confounders of this relation as depicted in Figure 8, traditional linear regression analyses would overestimate the hypothesized mediated effect. Sequential g-estimation produced the estimate of the mediated effect that was the closest to the true value of the mediated effect of transformational leadership on persistence though its effect on psychological needs. Although the doubly robust sequential g-estimation, IPW, and truncated IPW also resulted in overestimated mediated effects of transformational leadership on persistence through its effect on psychological needs, these estimators were closer to the true mediated effect than traditional linear regression and all included the true value of the mediated effect in the 95% confidence intervals except for doubly robust sequential g-estimation (see Table 1). Even in the case that the mediator model was misspecified (i.e., one measured confounder, identification, was left out of the analysis) sequential g-estimation provided the second to least biased estimate of the mediated effect of transformational leadership on persistence through its effect on psychological needs only behind IPW (see Table 2). Only the 95% confidence interval for the IPW estimate included the true value of the mediated effect when the mediator model was misspecified. When both the mediator and outcome models were misspecified, doubly robust sequential g-estimation resulted in similar bias as linear regression.

**TABLE 1:** Mediated effect estimates of simulated dataset for five correctly specified models for *N* = 500.

Models	True value	Estimate	LCL	UCL
Linear regression	0.759	1.236	0.946	1.579
Sequential *g*	0.759	0.785	0.587	1.016
Sequential *g* – doubly robust	0.759	1.012	0.765	1.297
IPW	0.759	0.952	0.623	1.278
IPW – truncated	0.759	1.091	0.736	1.440

**TABLE 2:** Mediated effect estimates of simulated dataset for seven misspecified models for *N* = 500.

Misspecified models	True value	Estimate	LCL	UCL
Linear regression	0.759	1.261	0.956	1.587
Sequential *g*	0.759	1.053	0.795	1.326
Sequential *g* – DR mediator misspecified	0.759	1.111	0.795	1.326
Sequential *g* – DR outcome misspecified	0.759	1.250	0.947	1.583
Sequential *g* – DR both misspecified	0.759	1.301	0.962	1.647
IPW	0.759	1.050	0.750	1.368
IPW – truncated	0.759	1.100	0.769	1.411

## Discussion

To test the research question of how robust the methods are to different confounder effect sizes and types (i.e., baseline versus post-treatment), models including two mediator-to-outcome confounders were estimated for each simulation condition (i.e., two-confounders estimation models). Below we present our discussion of the findings and would like to note that this study is limited to linear models, a limitation regarding the generalizability of the results.

The sequential g-estimation method is designed to handle post-treatment confounders, and the simulation results confirmed that it produced the most accurate estimates of the indirect effect in the presence of post-treatment confounders. Furthermore, results showed that IPW’s performance was mainly influenced by the confounder effect size. This may happen because as confounder effect size increases the variability of IPW estimates increase (Robins, 2000; Goetgeluk et al., 2008; Vansteelandt, 2009). This is also in line with a previous study which found that IPW did not work well as the effect size of baseline measures of *M* and *Y* increased in the case of a two-wave mediation model (Valente et al., 2019). Furthermore, in this study, we used a weighting strategy for the propensity score approach to causal mediation analysis; however, other strategies such as matching or stratification may have performed better (Rosenbaum and Rubin, 1984; Rubin and Thomas, 1992). Even though there is no study showing how matching would perform in the case of mediation analysis, propensity score studies addressing the *X* to *Y* relation indicate that matching may work better than weighting to achieve unbiased causal estimates (Frölich, 2004). Weighting estimates can be problematic since the estimates can be highly influenced by the assigned weights of individuals with propensity scores that are close to values 0 or 1 (Kang and Schafer, 2007; Schafer and Kang, 2008). In order to avoid extreme weights, the IPW method with truncated weights was also included in this study. However, results showed that in general, the IPW-truncated method did not perform better than the conventional IPW method. This finding may be due to the trimming rule used in this study (weights were trimmed at the 1st and 99th percentile of the weight distribution) and some other trimming strategies may yield better results. Simulation studies point out that trimming may optimize propensity score weights by decreasing variability in the weights, while the optimal level of trimming may be difficult to determine. Researchers should evaluate which trimming option best suits their data using evaluation criteria such as the least mean square error (Potter, 1993). However, other literature suggests that researchers should focus on correct specifications of their propensity score model rather than relying on trimming methods (Freedman and Berk, 2008; Lee et al., 2011).

Omitting one of the confounders from estimation corresponds to the common case where no measure of a confounder is available but a confounder may affect the analysis. So far, omitted variables bias has been largely investigated in the case of *X* to *Y* relationship (Antonakis et al., 2010). Methods such as difference-in-differences, propensity score analysis, regression discontinuity, and instrumental variables are recommended whenever researchers want to achieve causally interpretable estimates when *X* is not randomized. The causal mediation literature basically employs these methods to solve the non-randomized mediator status issue. For example, using the instrumental variable method, randomized treatment assignment (*X*) can be used as an instrument to mimic randomization for the *M* to *Y* relationship (Jo, 2008). However, this approach requires different assumptions such as the exclusion restriction that requires no relation of *X* on *Y* that is not through *M*. The exclusion restriction assumption means that there is not a direct effect of the instrumental variable on the outcome, which is difficult to satisfy in the mediation context. In this paper, we have focused on methods estimating controlled direct effects (i.e., IPW and g-estimation) to investigate omitted variable bias in mediation since we believe they are more realistic for social sciences research. Furthermore, different from the omitted variables literature in the context of *X* to *Y* relation, the causal mediation literature also focuses on the effect of omitting potential confounders that are influenced by the treatment (i.e., post-treatment confounders). Yet, the results of the current study point out that when models are misspecified by omitting one of the confounders from the analysis (i.e., one-confounder estimation model), failing to measure potential post-treatment confounder variables in a mediation model leads to biased estimates regardless of the analysis method used and emphasize the importance of sensitivity analysis for causal mediation analysis.

One aim of the current paper was to investigate the performance of doubly robust g-estimation, as it has been suggested in the literature as a superior method to g-estimation. In the case of two-confounders estimation model, the pattern of results for the doubly robust sequential g-estimation method’s performance was in general similar to the IPW method rather than sequential g-estimation. The doubly robust g-estimation method employed in this study used IPW estimation, and results suggest that the performance of the doubly robust method was influenced heavily by the IPW part of the estimation. Moreover, in the case of one-confounder estimation model, the doubly robust method had the highest bias when both parts of its estimation process (the propensity and outcome models) omitted the confounder *C*_2_. This finding was consistent with the warnings from the literature on the use of doubly robust methods (Schafer and Kang, 2008).

The focus of this article was on the causal identification of the indirect effect in the case of a randomized treatment. However, many studies in psychology involve non-experimental studies where *X*, *M*, and *Y* are all observed variables. When *X* is not randomized (or randomization fails), the researcher must adjust for all treatment-to-outcome confounders in order to satisfy the no unmeasured confounder for the *X* to *Y* (and *X* to *M*) relation assumption (VanderWeele and Vansteelandt, 2009). One potential solution to estimating causal indirect effects in the presence of *X* to *Y* confounders using the marginal structural model approach is described in VanderWeele (2015). Specifically, in addition to the weight for the mediator (*w*_i_^M^), a weight for the exposure (*w*_i_^X^) can also be created. The exposure weight will reflect the probability that each person would have received the treatment conditional on baseline covariates. Then, the product of these weights (*w*_i_^X^ × *w*_i_^M^) can be used to weight each individual when employing the marginal structural model approach to estimate direct and indirect effects.

## Recommendations

Based on the current study, the following recommendations for researchers can be offered.

(1) *When confounders for the M to Y relationship are measured, it is important to identify the types of confounders (baseline vs. post-treatment) in order to choose the analysis method to be implemented.* When confounders are measured and included in the analysis, linear regression with adjustment can be used for estimating the indirect effect; however, if the measured confounders are post-treatment, then sequential g-estimation is recommended. IPW should be used with caution as it leads to biased estimates as the confounder effect size gets larger.
(2) *Researchers should carefully consider the potential confounder variables for their mediation model when designing the study and make an effort to measure the confounder variables.* Our simulation study shows that when failing to accommodate confounder variables of the *M* to *Y* relationship in a mediation model with linear effects, all methods lead to biased estimates of the indirect effects, especially when the confounders are post-treatment.
(3) *Sensitivity analysis methods are highly recommended*. Sensitivity analysis methods can be used to evaluate how robust the indirect effect is to unmeasured third variables. In this case, even if important confounders are not measured and included in the analysis, some idea of the robustness of results to potential confounders can be obtained. It is also unlikely that researchers usually would have measured all potential confounders. Sensitivity analysis has been an important area of research to improve causality in treatment effects when randomization has not been possible (Rosenbaum and Rubin, 1983). For example, Cornfield et al. (1959) found that the relationship between smoking and lung cancer can be significantly weakened if a confounder variable for that relationship would be nine times more frequent in heavy smokers compared to non-smokers. Sensitivity analysis has been especially seen as an indispensable part of statistical mediation analysis (Imai et al., 2010a, b). Current literature suggests several sensitivity analysis methods for mediation analysis (Cox et al., 2013). For example, a technique described by VanderWeele (2010a) is based on the relation of the confounder to *Y* and the difference in the proportion of individuals with the confounder prevalence between the experimental groups at the same level of the mediator. Another method expresses confounder bias as the correlation between the error terms of the mediator and outcome regression equations (Imai et al., 2010a, b). Another method by Mauro (1990) is based on the correlations of a potential confounder with study variables. Statistical software code for the above-mentioned sensitivity analysis techniques is available in Imai et al. (2010b) and Cox et al. (2013). Furthermore, researchers can have access to the statistical software code for the investigated causal mediation methods at MacKinnon and Pirlott (2015).
(4) *Researchers can also improve the internal validity of their mediation model by choosing among several research designs that attempt to manipulate the mediator when it is both practically and ethically possible.* Double randomization design that involves the manipulation of the mediator enables a better causal interpretation of the *M* to *Y* relationship than the conventional measurement-of-mediation designs (Spencer et al., 2005; Stone-Romero and Rosopa, 2011; Pirlott and MacKinnon, 2016). A double randomization design randomly assigns participants to *X* and measure *M* and *Y* in experiment 1, and then, randomly assign participants to levels of *M* in a second experiment. If there is a significant effect of manipulated *M* on *Y* in the second experiment, it indicates a causal *M* to *Y* path. There are several types of mediator manipulations. Examples are the enhancement manipulation where exposure to the mediator is manipulated by enhancing the dose of the mediator, and the blockage manipulation in which the mediator is blocked in one condition but not in another condition to investigate if the effect of the treatment depends on the mediator (Robins and Greenland, 1992; Spencer et al., 2005; MacKinnon, 2008; Stone-Romero and Roposa, 2008; Bullock et al., 2010; Imai et al., 2013; Holland et al., 2016; Pirlott and MacKinnon, 2016).

Causal inference in the presence of mediating variables is an important area of research that has led to recent advancements, but there is a need for future work. For example, the sequential g-estimation method may be the preferred method in the presence of post-treatment confounders; however, it only allows the computation of a total indirect effect by obtaining an accurate direct effect estimate. Future research should extend causal mediation methods to more complex situations such as multiple mediators, longitudinal, and multilevel mediation models. For instance, even though applications in multilevel causal modeling exist, there is considerable need for analytical work and simulation studies (Hong and Raudenbush, 2006; Hong, 2010; VanderWeele, 2010b). Another important area of work for causal mediation is the development of alternative experimental designs in which researchers manipulate the mediator as described above. Future work is crucial in evaluating the advantages and disadvantages of the proposed experimental designs, clarifying the assumptions, and developing and illustrating the analysis of such designs. Such work in both experimental and quantitative approaches to mediation would encourage substantive researchers to apply causal mediation methods to real data.

Mediation analysis is an important tool to identify causal mechanisms of phenomena. Since mediation analysis, by nature, attempts causal claims, investigation of the best possible methods to estimate causal indirect effects has become an intensive area of research. This manuscript illustrates the performance of some of these modern techniques and provides guidance to implement them. The simulation study, by showing the distinct effects of baseline and post-treatment confounders on the accuracy of the estimates, concludes that when post-treatment confounders are omitted, estimates are biased. We are hopeful that this manuscript will improve the practice of mediation analysis and causal conclusions based upon it.

## Data Availability Statement

The raw data supporting the conclusions of this article will be made available by the authors, without undue reservation, to any qualified researcher.

## Author Contributions

YK-S, DM, and MV contributed to the conception of the overall method, conducted the simulation study, and manuscript writing. EÇ contributed to manuscript writing and editing. All authors read and approved the submitted manuscript.

## Conflict of Interest

The authors declare that the research was conducted in the absence of any commercial or financial relationships that could be construed as a potential conflict of interest.
